# Study of Machining of Gears with Regular and Modified Outline Using CNC Machine Tools

**DOI:** 10.3390/ma14112913

**Published:** 2021-05-28

**Authors:** Rafał Gołębski, Piotr Boral

**Affiliations:** Department Technology and Automation, Czestochowa University of Technology, Al. Armii Krajowej 21, 42-200 Częstochowa, Poland; piotrek@itm.pcz.pl

**Keywords:** CNC machining, involute, multipass method, outline, roughness, spur gear, surface integrity parameters

## Abstract

Classic methods of machining cylindrical gears, such as hobbing or circumferential chiseling, require the use of expensive special machine tools and dedicated tools, which makes production unprofitable, especially in small and medium series. Today, special attention is paid to the technology of making gears using universal CNC (computer numerical control) machine tools with standard cheap tools. On the basis of the presented mathematical model, a software was developed to generate a code that controls a machine tool for machining cylindrical gears with straight and modified tooth line using the multipass method. Made of steel 16MnCr5, gear wheels with a straight tooth line and with a longitudinally modified convex-convex tooth line were machined on a five-axis CNC milling machine DMG MORI CMX50U, using solid carbide milling cutters (cylindrical and ball end) for processing. The manufactured gears were inspected on a ZEISS coordinate measuring machine, using the software Gear Pro Involute. The conformity of the outline, the tooth line, and the gear pitch were assessed. The side surfaces of the teeth after machining according to the planned strategy were also assessed; the tests were carried out using the optical microscope Alicona Infinite Focus G5 and the contact profilographometer Taylor Hobson, Talysurf 120. The presented method is able to provide a very good quality of machined gears in relation to competing methods. The great advantage of this method is the use of a tool that is not geometrically related to the shape of the machined gear profile, which allows the production of cylindrical gears with a tooth and profile line other than the standard.

## 1. Introduction

It can be said with certainty that cylindrical gears are still the most frequently used gears. They are mainly processed by the hobbing method with modular hob cutters, as well as with Fellows chisels, or a finishing process by grinding according to the Maag, Niles, or Reishauer methods. Involute cylindrical gears with straight teeth are most commonly used in drive units in many machines and devices. They are most often machined using shaping, as presented by Litvin and Fuentes [1]. In addition, in unit production, they can be shaped with modular disc or finger cutters. The study of gear machining with modular disc cutters was presented in the work by Pasternak and Danylchenko [2], who simultaneously analyzed the distribution of cutting forces in the process.

The accuracy of the machined gear to the greatest extent depends on the technology and tool used, which can be guaranteed by the use of common tools such as modular hobs, while an even greater increase in the accuracy of these tools will allow for wider use of multipurpose universal CNC machine tools, as investigated by Piotrowski et al. [3]. These are special and usually expensive tools, and the machining is carried out on special machines.

The indicated technologies for the construction and design of gears were described in the works by Radzevich [4], Skoć and Switoński [5], and Nieszporek [6]. At present, there is a noticeable tendency to inspect manufactured gears using contactless methods; for example, the method of optical inspection of gears made of polymer using an injection method was presented by Urbas et al. [7].

Polymer materials are more and more often used as construction materials of gear elements. The polymer material itself can be modified by annealing in order to improve machining conditions with small allowances, as assessed by Gnatowski et al. [8]. Quite often, gears are machined with special tools, as presented in the work of Boral et al. [9] while analyzing the accuracy of the described method.

The use of a mathematical model in the process of designing the geometry of a special tool was presented in an article by Xin-Chun et al. [10], which can be the basis for optimizing the parameters of the milling cutter used to machine the gear. Gear machining technology is becoming increasingly demanding in terms of both quality and efficiency. It has also become very important that, in the manufacturing process, the machined gear does not have to undergo finishing processes on another machine tool. Thus, the done-in-one machining philosophy, i.e., the integration of all machining processes from clamping the raw material to finishing in a single machine, is increasingly becoming a priority [11]. The development of the done-in-one technology involves the integration of selective laser hardening with a multi-axis machining platform. This allows gears to be machined and hardened in one production center, as concluded by Peeters et al. [12].

One of the ways to improve the quality of gears after heat treatment is grinding, which is necessary in the finishing process; problems related to errors in the finishing process and their further consequences in the operation of the gears were presented in work by Gołębski and Szarek [13]. It is also possible to apply a new approach to the finishing of gears using non-Newtonian liquid polishing technologies, as described by Nguyen et al. [14]. The quality of the entire gearbox is determined by the location of the mating area of interacting elements, whereby mating analysis is mainly used to determine kinematic errors that are the result of machining and errors in gear assembly, optimization, and the synthesis of the gears (especially for the analysis of spatial gearing), as described in the work Nieszporek et al. [15].

The predictability of machining parameters, the characteristics of chip formation during machining, tool wear, and the generation of forces in the cutting process are currently the subjects of intensive industrial research, constantly contributing to the development of modern areas of gear machining, as presented by Bouzakis et al. [16]. With the increasing demand within the automotive industry around the world, a highly efficient method of gear manufacturing is required. Gear transmissions are used not only in cars but also in all kinds of mechanical products, including airplanes, ships, and power-generating equipment, and the production volume of gear boxes is huge. Álvarez et al. [17] investigated development in the field of design and construction of CNC machine tools allowing for the development of dedicated gear machining technologies, allowing for a free approach in terms of the shape and geometry of gears.

The advent of multitasking machines in the machine tool sector presents new possibilities for processing large-size gears in a single production on these machines. However, the possibility of using standard tools in conventional gear machining machines presents a technological challenge in terms of the quality of the product. However, the use of appropriate solutions and developing methods in CNC machining technology gear seems fully justified, as investigated by Skoczylas et al. [18]. One example of this is the power skiving technology as an efficient method for producing internal high-precision gears, as investigated by Inui et al. [19]. Klocke et al. [20] presented an analysis of the influence of geometrical parameters for the power skiving method on the quality of the manufactured gear. In the process of this machining method, a very important role is played by the visualization of the resulting machining shape, whereby we can precisely analyze the changes in the shape of the gear during the machining process, as well as the volume of the workpiece material removed, as noted by McCloskey et al. [21]. The method of machining according to the power skiving technology in the field of machining of internal gears is unmatched in terms of both quality and efficiency; however, machines dedicated to this method are very expensive, as presented by Chung-Yu et al. [22]. Software in which we can prepare the technology allows for the geometrical assessment of the machined gear by simulating the manufacturing process of a gear wheel, as pointed out by Spath et al. [23]. In contrast, Hyatta et al. [24] presented a very bold thesis that CNC machine tools with the use of appropriate technology of gear machining can offer a much higher productivity and quality level.

The possibility of generating the tool path and verifying the model of the machined part at the stage of technological preparation of production gives great opportunities for using the technology offered by computer control systems of machines, as presented by Yi et al. [25]. Karpuschewski et al. [26] presented an analysis of the surface integrity states for various variants of gear machining, concluding that the skiving milling method can compete with the finishing machining processes for small modular gears. Scherbarth’s [27] patented method of gear machining InvoMilling developed by DMG MORI and Sandvik using universal and simple tools represents a highly effective use of universal machining centers for productive and accurate gear manufacturing. Continuous development in the field of CNC control systems has created opportunities for technologists and programmers, leading to increasingly wider applications. Golebski [28] presented the advantages of parametric programming of CNC machine tools and the possibility of using this technology in the strategy of machining gears by the multipass method, while Jiang et al. [29] used the method of parametric programming to generate the NC code of gear machining for the case of machining with a modular hob. Another important idea in the whole process of using numerically controlled machines is that of using machine tools with the simplest kinematics, with particular emphasis on three-axis machine tools, which, in the case of machining straight gears, seems to be fully justified, as noted by Suh et al. [30].

A spur gear allows transmitting rotational movement and load adequate to its work. Therefore, the modification of the tooth line should be determined from the strength conditions of the gear. The first study on the optimization of the gear structure, with particular emphasis on the tooth profile, was conducted by Novikow, who proposed an innovative type of gear with a concave–convex profile, described in a study by Markowski and Batsch [31]. As a result of affixing, the trace of the mating teeth, modified under load, transforms into a point contact and should be located only in the area of the useful tooth surface. Spur gears with straight teeth with a longitudinally modified profile are characterized by insensitivity to assembly errors and the ability to transfer large loads. The assessment of the influence of the torsion angle of the axes on the mating area of gears and the position of the trace path of overlapping tooth was presented in work of Gołębski et al. [32], showing that, in the case of involute gears without longitudinal modification, the impact of errors in the gear assembly is significant for the mating area of gears.

According to the literature review, it can be stated with certainty that the topic taken up in the work is up to date and worthy of interest. It has been shown that gears can be machined using many methods, with particular emphasis on machining using CNC machine tools. The proposed method of machining cylindrical gears with straight teeth with longitudinal modification of the tooth line on universal CNC machines, using universal tools as cylindrical or spherical end mills, can represent an alternative to the industrial methods described in the work. It should be noted that the undertaken task is universal and may be further developed thanks to the application of a tool that is not geometrically related to the shape of the machined gear profile, which allows the production of cylindrical gears with a tooth and profile line other than the standard. In the context of the indicated features of the tested method, the analysis carried out in the work can answer the question of whether the accuracy of the developed method is adequate and can be used to produce the advisable types of gears.

## 2. Materials and Methods

The assumption of the method is to create an involute from tangential straight or spherical sections formed by the tool operating surfaces, similar to chiseling. In the machining process, the tool in the form of an end mill with a rectilinear or spherical contour enters the working position periodically, and then the tool contour is mapped on the workpiece. Next, the tool goes beyond the area of the workpiece and, during this time, a relative rolling motion takes place, consisting of a rotation in relation to the axis of the machined wheel, shifting parallel to the tool’s running line. The tool enters the working position and maps the contour of the machined gear tooth, but it is rotated with respect to the last machining trace. Since the machining traces are lines that are not parallel to each other, they intersect with each other, creating an angular transition between the tooth profile fragments. The machining cycle repeats itself, and the outline of the workpiece is actually angular. Therefore, the involute outline of the gear tooth is theoretical, which the angular contour follows when increasing the number of tool passes in the machining process to infinity. This section presents an analytical description of this method.

### 2.1. Involute Tooth Outline

**Figure 1:**
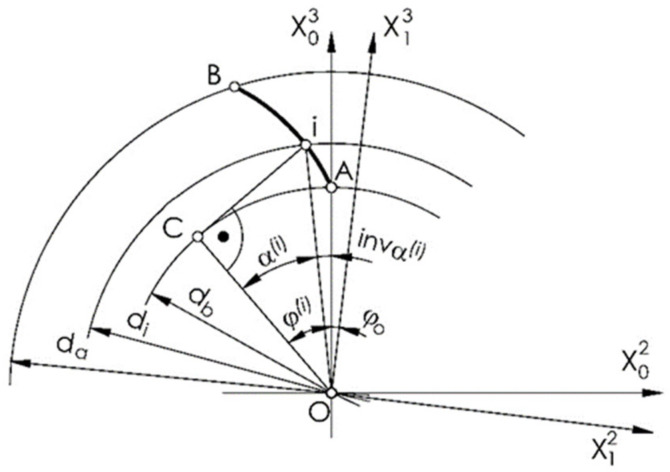
Tooth outline: left side of the tooth gap.

In order to machine the outline tooth of a gear using the multipass method, its individual points should be determined in the tooth outline coordinate system {0} with the axis of the ordinate passing through the starting point A of the outline of the wheel tooth (Figure 1).

(1) i=1…n.

The radii of successive points of the outline can be determined from the following equation (Figure 1):

(2) $$\frac{d_{i}}{2}=\frac{d_{b}}{2}+\frac{d_{a}-d_{b}}{2\left(n-1\right)}\left(i-1\right),$$

where $d_{i}$ is the diameter of the *i*-th point of the contour, and the angles of the outline are expressed as

(3) $$\alpha^{\left(i\right)}=\cos^{-1}\frac{d_{b}}{d_{i}},$$

where $\alpha^{\left(i\right)}$ is the profile angle at any point of the profile involute.

The angle between the ordinate axis and the leading radius of the point of tangency normal to the outline at this point with the base circle is expressed as follows (Figure 1):

(4) $$\varphi^{\left(i\right)}=\alpha^{\left(i\right)}+\mathrm{inv}\;\alpha^{\left(i\right)}=\tan\alpha^{\left(i\right)},$$

where $\varphi^{\left(i\right)}$ is the angle between the outline radius and the radius of the tangent point with the base circle of the outline normal $d_{i}$ at any point.

Lastly, the coordinates of any point of the tooth outline for the left side of the tooth gap in the {0} and {1} coordinate systems as shown in Figure 1 can be written as follows:

(5) $$x_{0}^{2\left(l\right)}=\frac{d_{b}}{2}\left(\sin(-\varphi^{\left(i\right)})+\varphi^{\left(i\right)}\cos(-\varphi^{\left(i\right)})\right),$$

(6) $$x_{0}^{3\left(l\right)}=\frac{d_{b}}{2}\left(\cos(-\varphi^{\left(i\right)})-\varphi^{\left(i\right)}\sin(-\varphi^{\left(i\right)})\right),$$

(7) $$x_{1}^{2\left(l\right)}=x_{0}^{2\left(l\right)}\cos\varphi_{o}-x_{0}^{3\left(l\right)}\sin\varphi_{o},$$

(8) $$x_{1}^{3\left(l\right)}=x_{0}^{2\left(l\right)}\sin\varphi_{o}+x_{0}^{3\left(l\right)}\cos\varphi_{o},$$

where, in $x_{*}^{o\left(*\right)},$ the subscript identifies the coordinate system, the index *o* (equal to 1 ∨ 2 ∨ 3) specifies the axis, and the index in parentheses indicates the outline (l ∨ r-left ∨ right) or the *i*-th point on the outline.

The gear tooth flank equation for the left side of the tooth gap has the following notation:

(9) $$r_{1}^{\left(l\right)}=r_{1}^{\left(l\right)}\left(\varphi ,\;u\right)=\left[u,\;\frac{d_{b}}{2}\left(-\sin\left(\varphi +\varphi_{o}\right)+\varphi \cos\left(\varphi +\varphi_{o}\right)\right),\;\frac{d_{b}}{2}\left(\cos\left(\varphi +\varphi_{o}\right)+\varphi \;\sin\left(\varphi +\varphi_{o}\right)\right)\right].$$

where *u* is a surface parameter.

Similarly, an outline for the other side of the tooth (right side of the tooth gap) can be written as follows:

(10) $$x_{0}^{2\left(r\right)}=\frac{d_{b}}{2}\left(\sin(\varphi^{\left(i\right)})-\varphi^{\left(i\right)}\cos(\varphi^{\left(i\right)})\right),$$

(11) $$x_{0}^{3\left(r\right)}=\frac{d_{b}}{2}\left(\cos(\varphi^{\left(i\right)})+\varphi^{\left(i\right)}\sin(\varphi^{\left(i\right)})\right),$$

(12) $$x_{1}^{2\left(r\right)}=x_{0}^{2\left(r\right)}\cos\varphi_{o}+x_{0}^{3\left(r\right)}\sin\varphi_{o},$$

(13) $$x_{1}^{3\left(r\right)}=-x_{0}^{2\left(r\right)}\sin\varphi_{o}+x_{0}^{3\left(r\right)}\cos\varphi_{o},$$

(14) $$r_{1}^{\left(r\right)}=r_{1}^{\left(r\right)}\left(\varphi ,\;u\right)=\left[u,\;\frac{d_{b}}{2}\left(\sin\left(\varphi +\varphi_{o}\right)-\varphi \cos\left(\varphi +\varphi_{o}\right)\right),\;\frac{d_{b}}{2}\left(\cos\left(\varphi +\varphi_{o}\right)+\varphi \;\sin\left(\varphi +\varphi_{o}\right)\right)\right].$$

### 2.2. Setting the Milling Cutter

**Figure 2:**
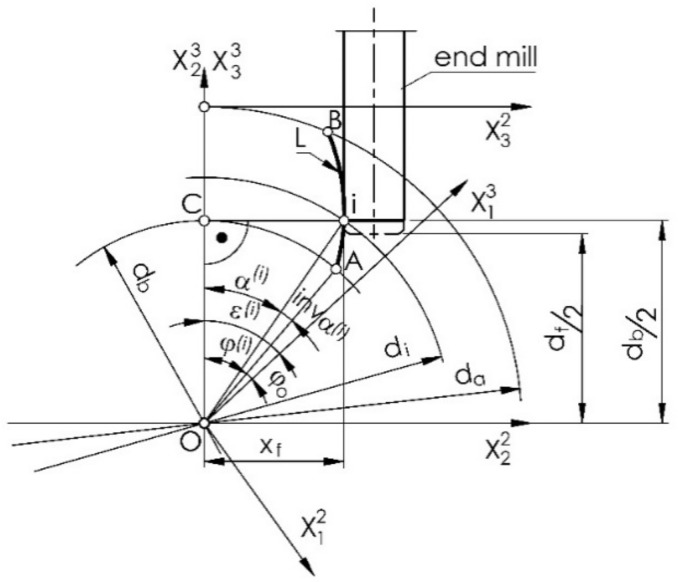
Setting the milling cutter during machining.

An involute outline is characterized in that the normals to it are tangent to the base circle. That is, it is possible to rotate the circle for the successive points of the outline (L. i) so that the contact points normal to the involute with the base circle (point. C) are always at the point of intersection of the base circle with the vertical ordinate (Figure 2). It follows that the end mill with a vertical axis, tangent to the outline being machined, for successive points is set at a constant distance from the horizontal axis of the gear, and only its displacement (offset) in the direction of the horizontal axis changes (its distance from the vertical axis of the wheel).

On a vertical milling machine, the cutter must be positioned within the radius of the base circle for each cut $\left(d_{b}/2\right)$ of the plane along the horizontal axis of rotation of the gear at the offset distance $\left(x_{f}\right)$ from the vertical axial plane of the gear, and the wheel must be rotated at an angle $\varepsilon^{\left(i\right)}$ (Figure 2).

The angle of rotation of the gear corresponding to the normal horizontal position of the tooth profile is equal to:

(15) $$\varepsilon^{\left(i\right)}=\varphi_{o}+\mathrm{inv}\;\alpha^{\left(i\right)}+\alpha^{\left(i\right)}=\varphi_{o}+\tan\;\alpha^{\left(i\right)},$$

where $\varepsilon^{\left(i\right)}$ is the gear rotation angle for machining the profile at the *i*-th point.

For the left tooth gap outline (right tooth outline), as shown in Figure 2, this can be expressed as follows:

(16) $$x_{2}^{2\left(il\right)}=x_{1}^{2\left(il\right)}\cos\varepsilon^{\left(i\right)}+x_{1}^{3\left(il\right)}\sin\varepsilon^{\left(i\right)},$$

(17) $$x_{2}^{3\left(il\right)}=-x_{1}^{2\left(il\right)}\sin\varepsilon^{\left(i\right)}+x_{1}^{3\left(il\right)}\cos\varepsilon^{\left(i\right)},$$

(18) $$x_{3}^{2\left(il\right)}=x_{2}^{2\left(il\right)},$$

(19) $$x_{3}^{3\left(il\right)}=x_{2}^{3\left(il\right)}-\frac{d_{a}}{2}=\frac{d_{b}}{2}-\frac{d_{a}}{2},$$

(20) $$\varepsilon^{\left(il\right)}=\varepsilon^{\left(i\right)}.$$

Similarly, for the outline of the tooth gap of the right tooth (left tooth profile), this can be expressed as follows:

(21) $$x_{2}^{2\left(ir\right)}=x_{1}^{2\left(ir\right)}\cos\varepsilon^{\left(i\right)}-x_{1}^{3\left(ir\right)}\sin\varepsilon^{\left(i\right)},$$

(22) $$x_{2}^{3\left(ir\right)}=x_{1}^{2\left(ir\right)}\sin\varepsilon^{\left(i\right)}+x_{1}^{3\left(ir\right)}\cos\varepsilon^{\left(i\right)},$$

(23) $$x_{3}^{2\left(ir\right)}=x_{2}^{2\left(ir\right)},$$

(24) $$x_{3}^{3\left(ir\right)}=x_{2}^{3\left(ir\right)}-\frac{d_{a}}{2}=\frac{d_{b}}{2}-\frac{d_{a}}{2},$$

(25) $$\varepsilon^{\left(ir\right)}=\varepsilon^{\left(i\right)}.$$

The outline of the tooth is symmetrical. The offset and the angle of rotation of the gear from a position where the axis of symmetry of the tooth gap is the vertical axis $x_{3}^{3}$ can be expressed as follows (the angle α is impractical because you cannot identify the beginning of the involute, but you can set the gear such that the tooth gap is symmetrically aligned with the vertical axis):

(26) $$x_{f}=x_{3}^{2\left(ir\right)}=-x_{3}^{2\left(il\right)},$$

(27) $$\varepsilon^{\left(r\right)}=\varepsilon^{\left(l\right)},$$

where $x_{f}$ is the milling cutter offset.

The starting position of the tool for machining the teeth across the entire width of the tooth was, thus, determined.

### 2.3. Shaping the Transitional Outline of a Tooth

**Figure 3:**
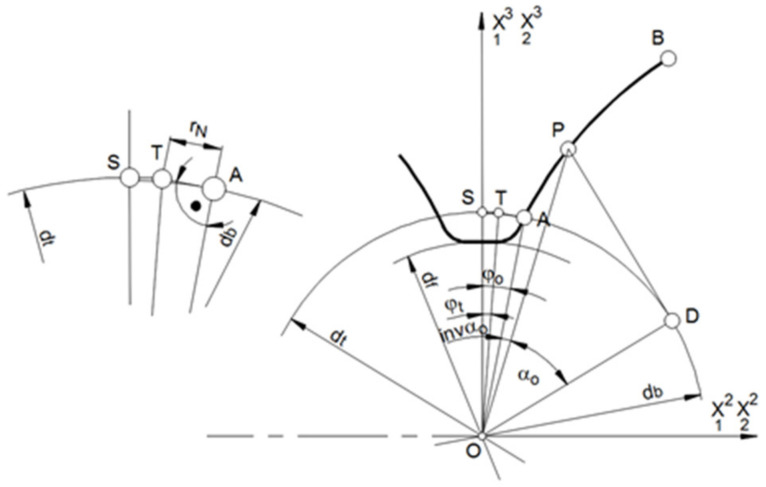
Shaping the transitional outline of a tooth.

The transition curve of the tooth profile side to the bottom land of the gear should be tangent to the involute profile at the outline starting point. Two methods can be used to machining this part of the tooth profile (this applies to machining below the base circle). In the first method, machining takes place automatically when making the main outline, assuming that the tool is positioned on the half of the tooth gap on the diameter of the base circle $\left(d_{f}/2\right)$ and is a ball end cutter with a radius equal to the fillet radius of the root circle. In the second method, it is possible to shape the transition outline with a milling cutter with a radius different from the contour of the tooth side. This method assumes that, in the machining of the transition curve, the gear does not rotate and the shaped tooth gap takes a symmetrical position in relation to the adopted coordinate system. The transition curve of the profile side may be different from the milling cutter radius (the milling cutter radius would be smaller; see Figure 3). The location of the point T of the cutter center for the machining of the tooth profile at point A of the involute profile depends on the tool radius, but the condition of not undercutting the other side of the fillet area must be achieved.

(28) $$r_{b}\tan\varphi_{o}\geq r_{N}.$$

The center of the milling cutter is set at successive points on the arc TS of the circle with a radius $r_{t}$ (Figure 3).

(29) $$r_{t}=\frac{d_{t}}{2}=\sqrt{\frac{d_{b}^{2}}{4}+r_{N}^{2}},$$

where $r_{N}$ is the milling cutter radius. Then, for the next points,

(30) j=1…m.

The position of the center of the tool sphere is determined by the angle

(31) $$\varphi^{\left(j\right)}=\varphi_{t}-\frac{\varphi_{t}}{\left(m-1\right)}\left(j-1\right),$$

where the angle defining the initial position of the center of the cutter sphere is equal to

(32) $$\varphi^{\left(i\right)}=\varphi_{o}-\mathrm{arc}\;\tan\frac{2r_{N}}{d_{b}}.$$

In the assumed case, the transition curve of the profile side into the bottom is a segment of a circle, and the bottom of the tooth gap is also a segment of a circle with a diameter

(33) $$\frac{d_{f}}{2}=\frac{d_{t}}{2}-r_{N}.$$

If we consider the Equations (33) and (29), then the cutter radius becomes equal to

(34) $$r_{N}=\frac{1}{4d_{f}}\left(d_{b}^{2}-d_{f}^{2}\right).$$

The possibility of shaping the transition outline with a milling cutter of a different diameter than the contour of the tooth side is an advantage of the described method, which is impossible in conventional machining technology.

### 2.4. Longitudinal Modification of a Gear Tooth

**Figure 4:**
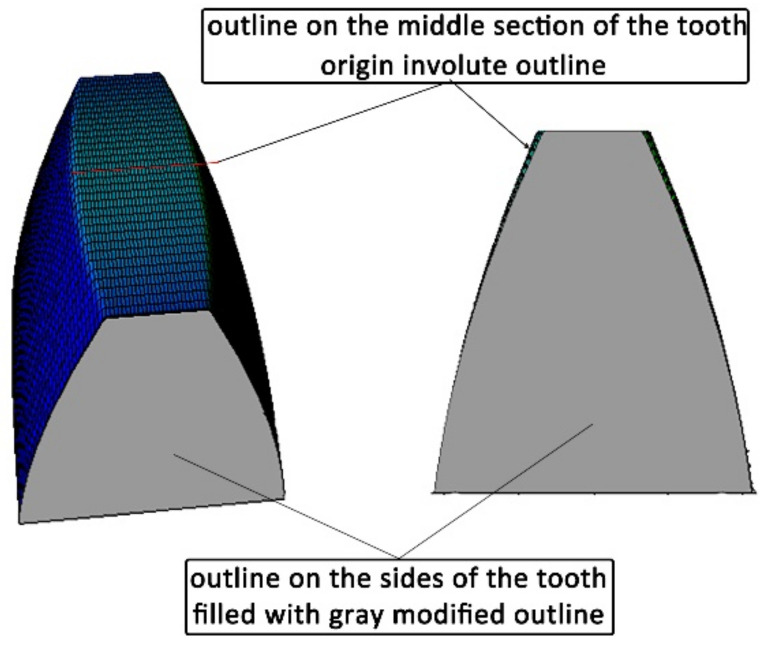
Longitudinal tooth line modification: calculation results.

**Figure 5:**
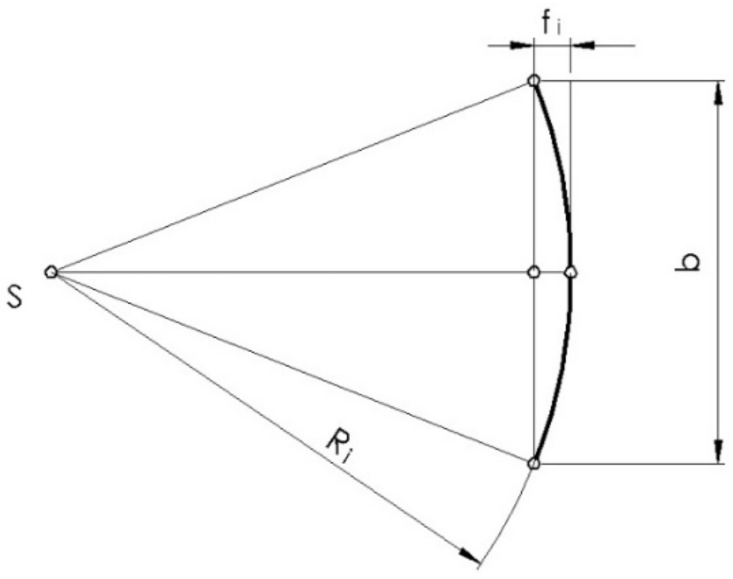
Scheme for determining the radius of the circle of longitudinal modification of a tooth.

The longitudinal modification consists of replacing the straight line forming the tooth at the considered point of the outline with an arc of a circle, the radius of which depends on the assumed modification at the ends of the tooth (see Figure 4 and Figure 5, where it is equal (at the tooth top land)).

(35) $$R_{min}=\frac{\frac{b^{2}}{4}+f_{max}^{2}}{2f_{max}},$$

where $f_{max}$ is the amount of longitudinal modification of the tooth at the top land, $R_{min}$ is the minimum modification radius of the longitudinal line of the tooth, and b is the gear rim width.

It can be assumed that the modification at the end of the sides of the tooth should not be less than the permissible deviation of the tooth line along the gear rim width, equal to the permissible error of axis non-parallelism. It was assumed that the longitudinal modification of the tooth may vary along the profile height from a value equal to zero at the starting point of the profile on the base cylinder to the assumed value on the outer diameter. This is due to the fact that the transition surface of the tooth side surface to the bottom land of the gear is not modified, and the transition of the tooth side to the bottom of the tooth gap would not be smooth (Figure 4).

As a result, the radius of the circle of the longitudinal modification of the profile changes for successive points along the height of the profile (Figure 5).

The circular interpolation function can be used to perform longitudinal modification of the gear tooth, where a radius is given or calculated by the control system. There is no modification at the starting point of the gear tooth profile, i.e., the G01 linear interpolation function is used at this point. However, at the next point of the outline, there is a minimum longitudinal modification resulting from the maximum value of the circular interpolation radius accepted by the machine tool control system.

(36) $$f_{min}=R_{max}-\sqrt{R_{max}^{2}-\frac{b^{2}}{4}},$$

where *f*_min_ is the amount of the minimum longitudinal modification of the tooth on the base circle, and *R_max_* is the maximum value of the radius of the circular interpolation.

Thus, in the subsequent points at the height of the outline, the longitudinal profile becomes

(37) $$R_{i}=R_{max}-\frac{R_{max}-R_{min}}{n-1}\left(i-1\right),$$

where $R_{i}$ is the amount of the modification radius of the longitudinal tooth line at the *i*-th tooth outline height.

### 2.5. Accuracy of the Outline

**Figure 6:**
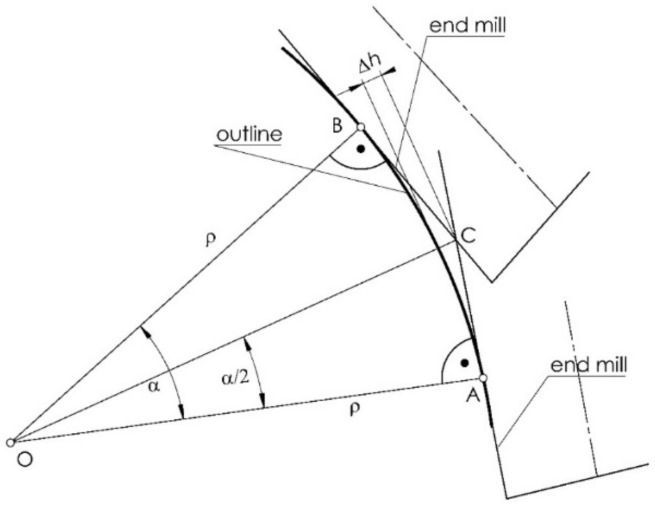
Scheme for determining the angularity error of the machined surface.

In the considered technology of gear machining, in simplified terms, for any convex outline, the curvilinear contour in the considered section is replaced by a straight section mapped by the tool. The end mill with a vertical axis during machining is a cylindrical surface tangent to the contour being machined. In general, the number of passes during finishing is large, whereby the distance between successive passes is small, i.e., a small section of the profile is processed, which can be considered as a straight-line segment. The maximum profile angularity can be determined according to the scheme presented in Figure 6, and the error of the profile angularity can be calculated.

(38) $$\Delta h=\frac{\rho }{\cos\frac{\alpha }{2}}-\rho ,$$

where Δh is the profile angularity error, ρ is the radius of curvature of the outline, and α is the difference in the angles of the tool settings with subsequent passes during machining.

To determine the estimated angularity of the tooth profile with the assumed method, the outline consists of a certain number of straight sections depending on the number of tool passes.

### 2.6. Program and Machining Process

**Figure 7:**
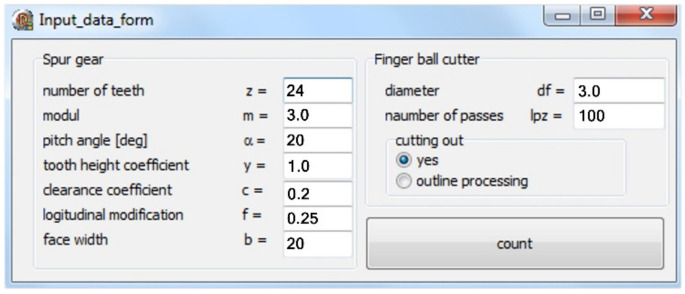
Software module: data introduction.

**Figure 8:**
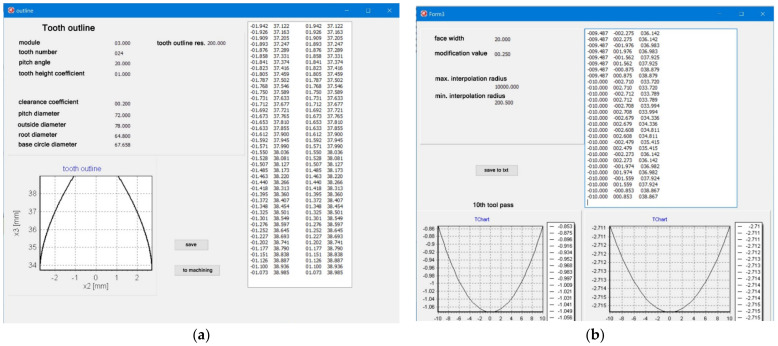
Software module: (**a**)tooth outline-generating software; (**b**)tool path-generating software.

On the basis of the analysis, applications supporting the generation of the coordinates of the modified outline and, consequently, of the tool path for the gear machining were developed (Figure 7 and Figure 8).

The software consists of two modules. In the first module, for the basic parameters of the gear *z* = 24, *m* = 3, *α* = 20°, *c* = 0,2, *x* = 0, *y* = 1 and the assumed longitudinal modification *f* = 0.25 mm, a discrete record of the coordinates of the modified outline was generated (Figure 8a), on the basis of which a solid model of the modified tooth was built to verify the correct operation of the software (see Figure 4).

At the next stage, the software generating the tool path consistent with the previously calculated outline was developed (Figure 8b). The entire generated program includes machine control functions in accordance with the ISO code. It is universal in that it can be adapted to any machine tool that can be programmed according to standardized functions. Depending on the parameters of the machine control system, it is important to limit the maximum radius of the outline modification. Analytical calculations show that the maximum radius tends to infinity; however, it seems justified in this situation to limit this value. In our case, the maximum interpolation radius was assumed at the level of 9999 mm. The generated toolpath is saved in a text file and can be merged with the code of the main program of the indicated control system. Two gears with different geometrical parameters were processed; the gear with the *m* = 3 mm module underwent longitudinal modification of the tooth line (Table 1).

**Figure 9:**
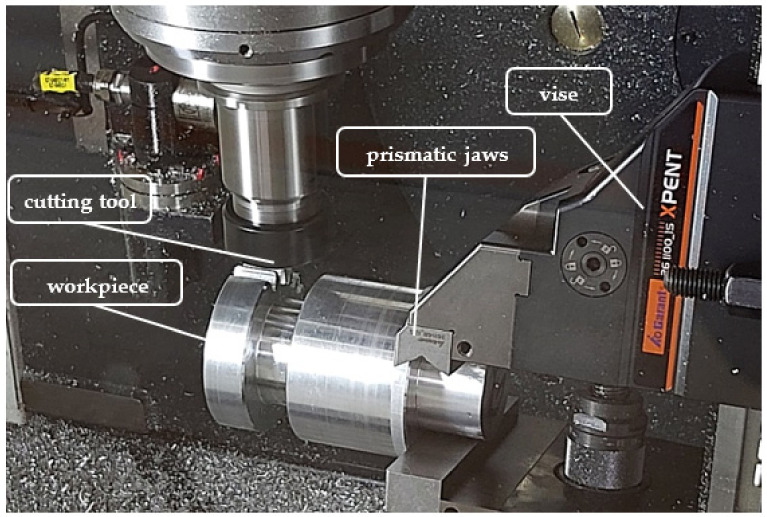
Clamping system in prismatic jaws: machining process.

A five-axis milling machine by DMG MORI, model CMX 50U (Pleszew, Poland), with a Sinumerik 840Dsl control system (version 4.7 Ed.4 SP6, 2020, Siemens, Munich, Germany), was used for machining; the control system was also equipped with the ShopMill programming overlap allowing for dialogue programming of the machine. The structure of the developed program included the integration of the generated ISO code, i.e., the paths of the tooth gap machining program with the basic program of the control format for Sinumerik (version 4.7 Ed.4 SP6, 2020, Siemens, Munich, Germany). In both cases, the blank was a uniform material, 16MnCr5 steel, with one end recessed to the diameter of ϕ50, enabling the blank to be fixed in the prismatic jaws of a vise (Figure 9).

**Figure 10:**
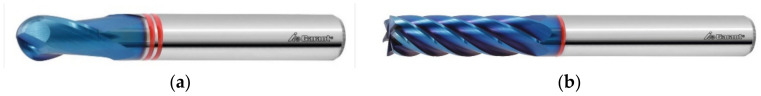
Solid carbide milling cutters used in processing: (**a**) ball end; (**b**) cylindrical [33].

A very important stage in the preparation of the machine tool for machining was the correct positioning of the workpiece, while maintaining the appropriate parallelism and perpendicularity of the surface. For this purpose, a Renishaw RMP 40 (Renishaw, New Mills, UK) wireless measuring probe was used to measure the workpiece to verify the correct mounting, i.e., the parallelism of the gear face to the table plane. In this case, the maximum deviation of the surface parallelism did not exceed 0.003 mm. The tool used for machining was a VHM ball-end cutter used for machining a gear with modification (machining with the spherical part of the tool) and a cylindrical cutter for machining a gear with a straight tooth line (machining with the cylindrical part of the tool). Tools had a diameter of ϕ3 mm and ϕ6 mm, with high accuracy, and they were made entirely of HPC sintered carbides with a titanium nitride coating with a thickness of 7 μm, for better chip evacuation (see Figure 10). The tool is adapted to work without cooling; during machining, the chips were removed with compressed air.

**Figure 11:**
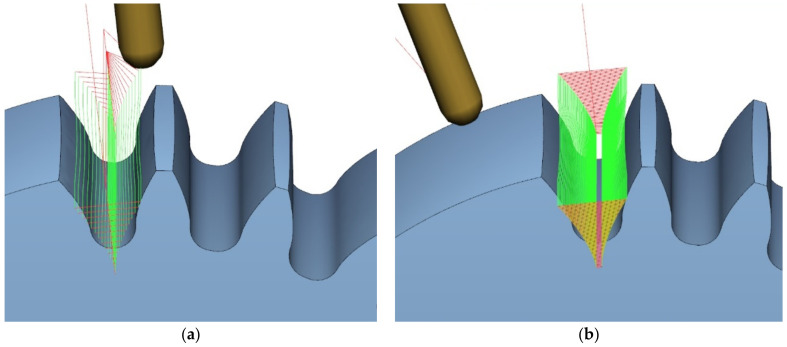
Adopted machining strategy: (**a**) roughing; (**b**) finishing.

The ball-end milling cutter, had two blades at the helix angle of 45°, while the cylindrical cutter had five blades, with a blade diameter tolerance not exceeding 10 μm; the maximum permissible contact width between the tool with a diameter of ϕ3 and the workpiece was 0.6 mm. The tool shank was made in accordance with standard DIN 6535 HA [34] with h5 tolerance. A tool holder was used, made in accordance with standard ISO 7388-1 [35], type ER32 SK40 A100, keeping the accuracy of the rotational movement ≤3 µm and the balancing accuracy of G 2.5 at a rotational speed of 25,000 rpm. The developed machining program included roughing (i.e., a plunge division of the tooth gap at the level of 18 layers; Figure 11a), after which an allowance for finishing was left, amounting to 0.12 mm distance from the outline. The finishing allowance was removed with tool passes in 85 layers (Figure 11b). The same tool was used for roughing the entire process. In the case of finishing, four of the same tools were used. Such assumptions were to limit the impact of the tool wear factor on the surface condition after machining.

## 3. Results

**Figure 12:**
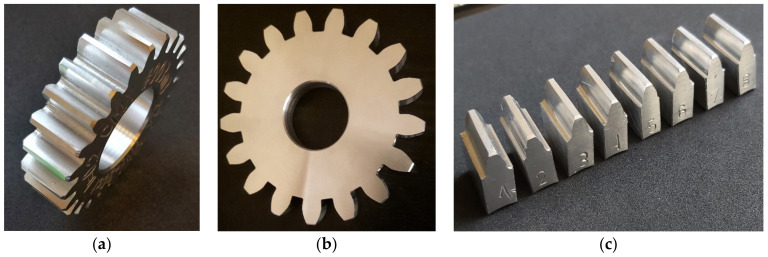
Machined gears (**a**) with modification *m* = 3, and (**b**) without modification *m* = 6; (**c**) tooth samples from modified gear.

Machined gears (Figure 12a,b) were tested for geometric parameters on a coordinate measuring machine, after which individual sections of the wheel rim could be subjected to surface tests after machining; eight teeth were cut out (Figure 12c). This solution ensured clear access to the surface during the measurement.

### 3.1. Measurement of Geometrical Compliance of a Gear with the Use of Software Module ZEISS Gear Pro Involute

**Figure 13:**
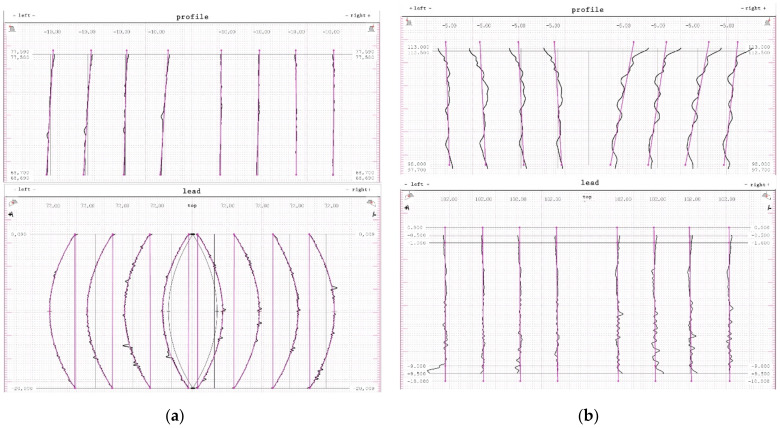
Graphical presentation of measurement results: (**a**) gear with tooth line modification, module *m* = 3; (**b**) gear with straight tooth line, module *m* = 6.

When manufacturing gears, achieving small tolerances of mating teeth is one of the most important ways to keep them quiet and lossless. Along with the increase in the accuracy of machine tools for machining gears, the methods of checking and measurement had to be improved as well. The manufactured gears were measured on a coordinate measuring machine using the Gear Pro Involute module of the ZEISS CALYPSO software (Version ZEISS CALYPSO 2020, 2020, Jena, Germany). By introducing the basic parameters of gears to the software module, an analytical 3D model was created on the basis of which the measurements were carried out. The following parameters of the gear wheel were determined during the measurements: Fα profile deviation, total overlay of profile form deviation and profile slope deviation, f_fα_ form deviation of the profile without consideration of the slope deviation, f_Hα_ profile slope deviation of the profile without consideration of the form deviation, Fβ tooth line (lead) deviation, total overlay of lead form deviation and lead slope deviation, f_fβ_ form deviation of the lead without consideration of the slope deviation, f_Hβ_ lead slope deviation of the outline without consideration of the form deviation, and C_β_ modification crowning correction of the helix through convex curvature over the face width measured on the pitch circle. Table 2 shows the tolerances of the indicated measurements as a function of the averaged results of the measurements of the whole gear. The measurement results are shown in Figure 13 on the graphical measurement report.

### 3.2. Measurement of the Structure of the Tooth Surfaces

**Figure 14:**
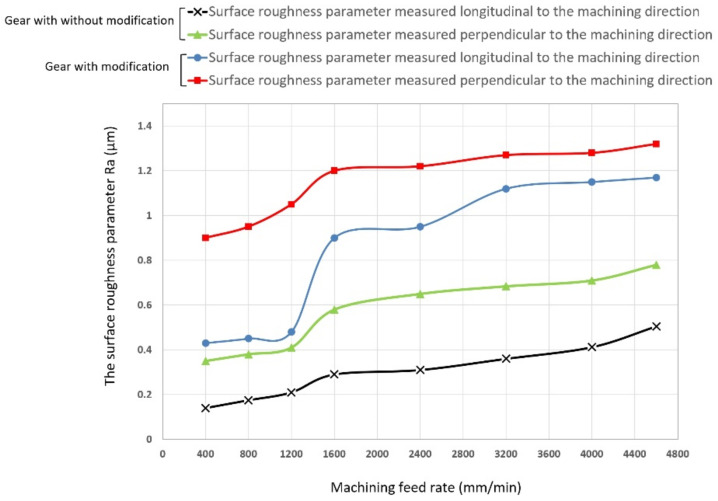
Measurement results: surface roughness Ra parameter, measured longitudinal and perpendicular to machining direction.

In the next stage of the research, the machined gear with a modified contour was subjected to a detailed analysis using the three-dimensional optical system of the Alicona Infinite Focus G5 microscope (Alicona Imaging GmbH, Raaba, Austria). A microscope is an excellent device for assessing the quality of the surface after treatment and for measuring roughness parameters. The toothed wheel with the *m* = 6 mm module was assessed on a laboratory contact profilographometer Taylor Hobson, Talysurf 120 (Taylor Hobson, New Star Road, UK). In both cases, the roughness of the machined tooth surfaces was measured in the direction of the contour in the middle of the rim width and along the tooth line at the height of the pitch diameter (Figure 14).

**Figure 15:**
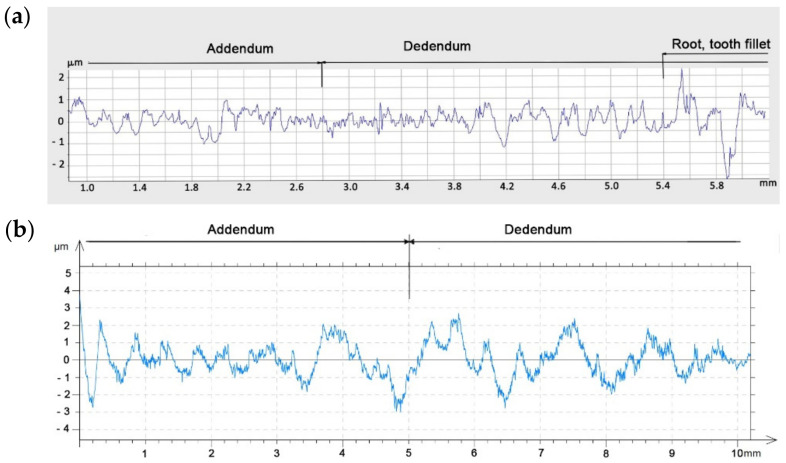
Roughness parameter profile Ra measured perpendicular to the machining direction: (**a**) sample 1, tooth with modification; (**b**) sample 1, tooth without modification.

**Figure 16:**
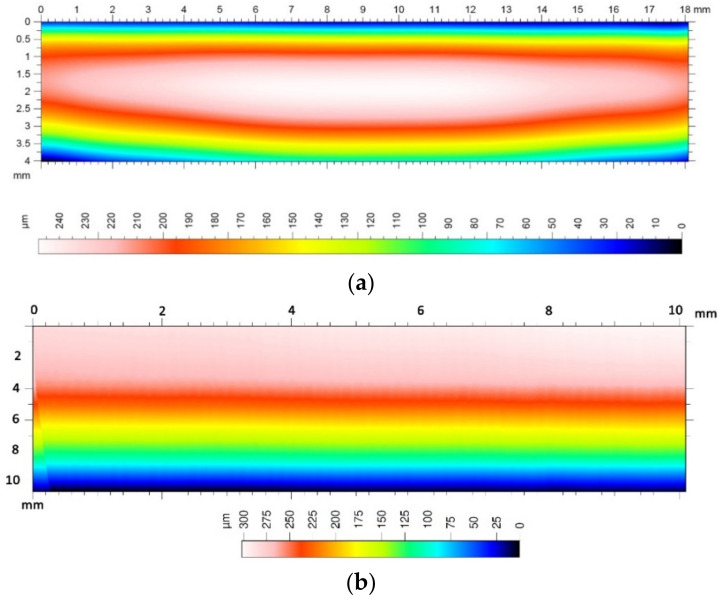
The stereometric distribution of the tooth surface: (**a**) sample 1, tooth with modification; (**b**) sample 1, tooth without modification.

Measurement of surface roughness parameters in the direction perpendicular to the tooth line is important due to the adopted machining strategy in the multipass method. It allows answering the question of whether the adopted division of cutting layers is appropriate. The measurement results in the direction parallel to the tooth line significantly depend on the adopted cutting parameters. Determination of the optimal feed speed allows increasing the productivity of the method without loss of quality of the treated surface. The microgeometric structure of the top layer was also assessed, whereby the measurement was made in the middle section of the tooth perpendicular to the processing direction (Figure 15). The stereometric distribution of the tooth convex area was also determined (Figure 16).

**Figure 17:**
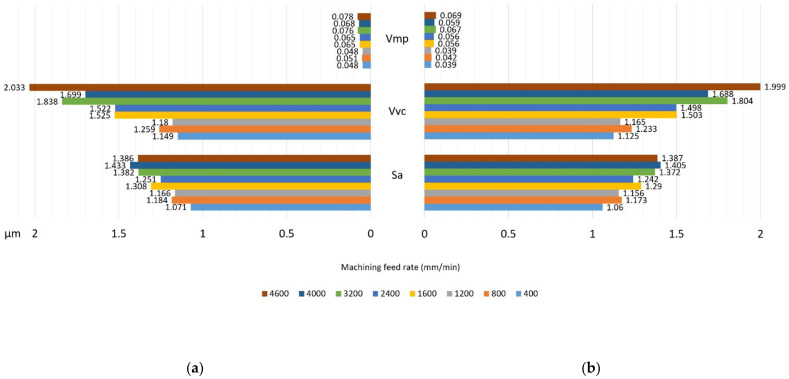
Selected surface roughness parameters (Sa, Vvc, and Vmp): (**a**) gear with modification; (**b**) gear without modification.

Figure 17 show selected surface roughness parameters measured perpendicular to the machining direction in the middle section of the spur gear. The following parameters were determined: Sa arithmetical mean height of the scale-limited surface, Vvc core void volume of the scale-limited surface, and Vmp peak material volume of the scale-limited surface. The parameters Vmp and Vmp allow a better understanding of the quality of the treated surface.

The Vvc parameter represents the difference in levels at which the void volume is determined with respect to the volume of the core material in the range of 10% to 80%. In contrast, Vmp is the level of the material volume to the void volume in the range from 0% to 10%, above the plane defining the core.

## 4. Discussion

From the point of view of the assessment of the operational properties of the gear, a very important research result was the evaluation of the gear parameters using the specialized Gear Pro Involute software. According to the ISO system of flank tolerance classification [36], on the basis of the obtained deviations (Table 2), we can determine the accuracy class of machined gears. Based on the parameters Fα profile deviation, total overlay of profile form deviation and profile slope deviation, ffα form deviation of the profile without consideration of the slope deviation, and f_Hα_ profile slope deviation of the profile without consideration of the form deviation, we can conclude that the obtained profile deviations classify the modified gear wheel with the module *m* = 3 as accuracy class 7. On the other hand, the gear without modification, module *m* = 6, can be classified as accuracy class 8. When assessing the tooth line on the basis of the obtained parameter results for Fβ tooth line (lead) deviation, total overlay of lead form deviation and lead slope deviation, ffβ form deviation of the lead without consideration of the slope deviation, and f_Hβ_ lead slope deviation of the outline without consideration of the form deviation, the gear wheel modified with the *m* = 3 module was assessed as belonging to accuracy class 7, while the wheel without modification with a straight tooth line was assessed as belonging to accuracy class 6. The outline in Figure 13 is correct; for a tooth modified in the axial section of the tooth, the involute line does not have excessive deviations. The image of the stereometric distribution of modifications in Figure 16 is symmetrical, which proves the correct positioning of the blank and the machining process itself. The deviations obtained may to a large extent result from the stiffness of the tool itself and the cutting forces occurring in the machining process. From the point of view of the efficiency of the tested methods and the effect of changing the machining parameters on the condition of the tooth flanks, it was important to determine the qualitative parameters of the surface assessment. Roughness measurements were made longitudinal and perpendicular to the tool path. Within the scope of dedicated normative feeds, the obtained results of the Ra parameter of the surface roughness did not exceed 1.2–1.6 μm (see Figure 14). The threefold increase in feed did not contribute to a deterioration of the machined surface. We can also say that machining with high feeds brings a number of benefits in this case; the volumes of the cut layers are very small and the use of high feed speeds causes the heat to be removed to an increasing extent together with chip. The microgeometric structure of the layer in the middle section of the tooth perpendicular to the processing direction in the addendum and dedendum areas showed an equally determined character (Figure 15). Only in the case of the tooth fillet area did deterioration occur excessively. It follows that, in this area for a spherical tool, the area of the tool contact zone increases, which may be accompanied by vibrations. This area is not actively involved in the mating of pairs of teeth. It seems justified, in this case, to machine the tooth fillet area with an additional tool of a smaller diameter or to make a geometric modification of the root area. Figure 17 shows the selected surface roughness parameters measured perpendicular to the machining direction in the middle section of a spur gear; the indicated parameters allow for more precise determination of the operational indicators of the tested surfaces, which allows concluding about the resistance of a given surface to tribological wear. In the case of a high value of the Vpm parameter, the surface is characterized by large elevations, which means that, in cooperation with another surface, the contact area is small and is associated with the occurrence of high unit pressure. The Vvc parameter illustrates the surface transmitting pressure after a given element is reached and the manner of wear of the mating surfaces, as well as the speed of formation of tooth gap clearances and size. These parameters are satisfactory; thus, after lapping, the operation of the transmission would be characterized by even wear and stable operation.

## 5. Conclusions

In summary, it can be stated that the multipass method indicated in the work has a number of advantages over conventional methods. Machining with a tool that is not geometrically related to the outline to be machined opens up many possibilities for gear design in the geometrical areas of its modification. It allows freely modifying the fillet area in order to, for example, improve its strength considerations. In the process of designing a wheel, one can also choose its parameters freely; using the same tool, one can process wheels with a wide range of modules, as well as nonstandard ones. Industrial software for manufacturing gears for CNC machine tools offered by leading manufacturers such as Sandvik Coromant or DMG MORI is very expensive and dedicated to machine tools with complex operating kinematics. The presented work proves that it is possible to machine gears with straight teeth, as well as those modified on a basic CNC machine tool, and the obtained results of geometric and surface accuracy are fully satisfactory. In the future, the presented machining methods can not only allow for the production of cylindrical gears with the modification of the tooth line, but also provide technological possibilities for the production of gears with a tooth line other than straight or arc. This will allow the construction of cylindrical gears with any shape of the tooth line and the possibility of their use in gear transmission.

## Figures and Tables

**Table 1:** Machined gears parameters.

Module (mm)	Number of Teeth	Pitch Angle (°)	Tooth Height Coefficient	Longitudinal Modification (mm)	Face Width
3	24	20	1.0	0.25	20
6	17	20	1.0	0	12

**Table 2:** Measurement results: tolerance fields.

Gear Type, Module (mm)	Avg. Fα (μm)	Avg. ffα (μm)	Avg. fHα (μm)	Avg. Fβ (μm)	Avg. ffβ (μm)	Avg. fHβ (μm)	Abs. C_β_ (μm)
with modification module 3	14	8	11	19	16	2	74
without modification module 6	22	15	11	8	9	2	-

## Data Availability

Data is contained within the article.
